# Primary Care Physicians’ Experiences With and Adaptations to Time Constraints

**DOI:** 10.1001/jamanetworkopen.2024.8827

**Published:** 2024-04-30

**Authors:** Michelle-Linh T. Nguyen, Vlad Honcharov, Dawna Ballard, Shannon Satterwhite, Aoife M. McDermott, Urmimala Sarkar

**Affiliations:** 1 National Clinician Scholars Program, University of California, San Francisco; 2 Center for Vulnerable Populations, Zuckerberg San Francisco General Hospital, San Francisco, California; 3 Department of Communication Studies, University of Texas at Austin, Austin; 4 Department of Family and Community Medicine, UC Davis Health, Sacramento, California; 5 School of Public Health, University of California, Berkeley; 6 Aston Business School, Aston University, Birmingham, UK; 7 Division of General Internal Medicine, Department of Medicine, University of California, San Francisco

## Abstract

**Importance:**

The primary care workforce shortage is significant and persistent, with organizational and policy leaders urgently seeking interventions to enhance retention and recruitment. Time constraints are a valuable focus for action; however, designing effective interventions requires deeper understanding of how time constraints shape employees’ experiences and outcomes of work.

**Objective:**

To examine how time constraints affect primary care physicians’ work experiences and careers.

**Design, Setting, and Participants:**

Between May 1, 2021, and September 31, 2022, US-based primary care physicians who trained in family or internal medicine were interviewed. Using qualitative analysis of in-depth interviews, this study examined how participants experience and adapt to time constraints during a typical clinic day, taking account of their professional and personal responsibilities. It also incorporates physicians’ reflections on implications for their careers.

**Main Outcomes and Measures:**

Thematic analysis of in-depth interviews and a measure of well-being (American Medical Association Mini-Z survey).

**Results:**

Interviews with 25 primary care physicians (14 [56%] female and 11 [44%] male; median [range] age, 43 [34-63] years) practicing in 11 US states were analyzed. Two physicians owned their own practice, whereas the rest worked as employees. The participants represented a wide range of years in practice (range, 1 to ≥21), with 11 participants (44%) in their first 5 years. Physicians described that the structure of their work hours did not match the work that was expected of them. This structural mismatch between time allocation and work expectations created a constant experience of time scarcity. Physicians described having to make tradeoffs between maintaining high-quality patient care and having their work overflow into their personal lives. These experiences led to feelings of guilt, disillusionment, and dissatisfaction. To attempt to sustain long-term careers in primary care, many sought ways to see fewer patients.

**Conclusions and Relevance:**

These findings suggest that organizational leaders must align schedules with work expectations for primary care physicians to mitigate physicians’ withdrawal from work as a coping mechanism. Specific strategies are needed to achieve this realignment, including incorporating more slack into schedules and establishing realistic work expectations for physicians.

## Key Points

**Question:**

How do primary care physicians experience and adapt to time constraints?

**Findings:**

In this qualitative study, physicians described a mismatch between the structure of their work hours and the work expected of them, which created constant time scarcity. To mitigate work overflow, physicians made tradeoffs in patient care and their personal lives, leading to guilt, disillusionment, and dissatisfaction; to sustain careers, many sought ways to see fewer patients.

**Meaning:**

Organizations must align schedules with physicians’ workload by introducing flexibility and establishing realistic work expectations to address the workforce shortage.

## Teaser

This qualitative study of US-based primary care physicians who trained in family or internal medicine examines primary care physicians’ experiences with and adaptations to time constraints.

## Introduction

A healthy, sustainable primary care workforce is necessary to ensure high-quality care, yet there has been a shortage of primary care physicians (PCPs) since World War II, with no sign of improvement.^1,2^ Few trainees are interested in pursuing careers in primary care,^3,4^ and many PCPs are experiencing exhaustion and burnout and considering changing careers.^5,6,7^ Time constraints due to time-related organizational policies (ie, work schedules) are not only known drivers of these undesirable workforce outcomes^8,9,10^ but also associated with negative changes to care quality.^11,12,13^ However, the underlying relationships between organizational scheduling policy, time constraints, care quality, and workforce well-being are complex. We need a more nuanced understanding of these relationships to create interventions that will promote a sustainable primary care workforce alongside careful, kind, and high-quality patient care. We initiated this work by using qualitative methods to extend understanding of PCPs’ experiences with and adaptations to time constraints.

## Methods

The design of this qualitative study was informed by the experiences of 2 of the authors (M.-L.T.N. and U.S.) working as PCPs in an academic safety-net setting and the economic and cognitive science theory of scarcity^14^—the subjective experience of having less than you feel you need. Within this framework, scarcity can increase focus on an immediate task, but it leads to behaviors that make us shortsighted. Although strategies born in scarcity often appear more efficient initially, scarcity ultimately slows the pace of real work (eg, by leading to more errors).^14^ By exploring the concept and theory of time scarcity through our interviews, we hoped to better understand the human experience of time constraints, including whether and how they impact primary care practice. The University of California, San Francisco Institutional Review Board reviewed and approved this study. The study followed the Standards for Reporting Qualitative Research (SRQR) reporting guideline.^15^

Between May 1, 2021, and September 30, 2022, we interviewed US-based PCPs who trained in family or internal medicine. We included only physicians who practiced at least 50% full-time equivalent clinical work and had been practicing primary care medicine for more than 1 year after residency training. We purposefully sampled a wide range of practice settings and clinical experience levels until thematic saturation was achieved. We invited physicians to participate by email. Virtual study visits were scheduled using Calendly software (Calendly), text, or email.

During the study visits, we collected demographic and practice characteristics as well as data from a measure of well-being (American Medical Association Mini-Z survey^16^ adapted from the Mini-Z 1.0 survey^17^ [eAppendix 1 in Supplement 1]). Demographic survey options were determined by the research team, including categories for self-reported race and ethnicity, which supported recruitment of a representative sample of physicians. Semistructured interviews between 45 and 60 minutes in length were conducted via Zoom video conferencing technology (Zoom Video Communications) to explore how participants experience and adapt to time constraints during a typical clinic day (M.-L.T.N.). The interviews were recorded with the verbal consent of all participants, including consent to present and publish deidentified quotations. All recordings were deidentified and professionally transcribed by a transcription service. Participants were compensated for their time with $100 gift cards to either Amazon or DoorDash.

### Data Analysis

We summarized the demographic and Mini-Z survey data to characterize our participant group. We then conducted a thematic analysis of interview data using a deductive and inductive coding process. We based our deductive coding on preestablished themes we prospectively explored through our interview questions (ie, test and referral ordering changes) (see eAppendix 2 in Supplement 1 for full interview guide), whereas inductive coding was used to take account of novel themes that emerged (ie, long-term implications for career sustainability). We underwent a group consensus process to ensure intercoder consistency: 5 of the same interview transcripts were coded using Dedoose software, version 9.0.18 (Dedoose) (M.-L.T.N. and V.H.), after which we held group meetings to discuss and review differing coding applications until consensus was achieved. All coding was then completed (V.H.). A detailed review and annotation of all transcripts was performed (M.-L.T.N.), and common themes across all transcripts were independently searched for and jointly discussed.

## Results

Twenty-five PCPs (14 [56%] female and 11 [44%] male; 7 [28%] Asian, 1 [4%] Black or African American, 16 [64%] White, and 1 [4%] race not reported; median [range] age, 43 [34-63] years) practicing in 11 states (Arizona, California, Colorado, Georgia, Illinois, Montana, New York, North Carolina, Pennsylvania, Texas, and Utah) volunteered to participate in this study. Two PCPs owned their own practice, and the rest were employees of organizations. The participants represented a wide range of years in practice (range, 1 to ≥21), with 11 participants (44%) in their first 5 years. Eighteen (72%) reported that their practice accepts Medicaid insurance. Our participants represented a wide range of practice experience with a predominance of physicians in their early careers (Table 1).

**Table 1.** Participant Characteristics

Characteristic	No. (%) of participants (N = 25)
Gender	
Male	11 (44)
Female	14 (56)
Race	
Asian	7 (28)
Black or African American	1 (4)
White	16 (64)
Not reported	1 (4)
Ethnicity	
Not Latino or Hispanic	24 (96)
Prefer not to answer	1 (4)
Age group, y	
30-39	9 (36)
40-49	7 (28)
50-59	3 (12)
≥60	2 (8)
Not reported	4 (16)
Accept Medicaid patients	
Yes	18 (72)
No	5 (20)
I don’t know	2 (8)
Type of residency training	
Family medicine	12 (48)
Internal medicine	13 (52)
Region of practice (US)	
West, excluding California	6 (24)
California	9 (36)
South	3 (12)
Midwest	2 (8)
Northeast	4 (16)
Not reported	1 (4)
Years in practice	
1-5	11 (44)
6-10	3 (12)
11-15	4 (16)
16-20	2 (8)
≥21	5 (20)

The median overall Mini-Z survey score was 30 (range, 21-37). Most participants were satisfied with their current job (median, 4 [agree]; range, 1-4). Most participants felt a great deal of stress because of their job (median, 4 [agree]; range, 1-5). Most endorsed 1 or more symptoms of burnout. See eAppendix 3 in Supplement 1 for a full summary of survey results.

A notable characteristic of physicians in this sample was an expressed passion for patient care and/or the future of their profession. For example, one participant summarized:

I love primary care and I am very appreciative that you are taking this step to know…what can be done to make our lives and our patients’ lives better…because I don’t want people to hate primary care, and I see that in new people coming up.

Our major findings center around physicians’ experiences of time, the ways in which they adapt and cope, and how they try to sustain clinical careers (Figure).

**Figure.**
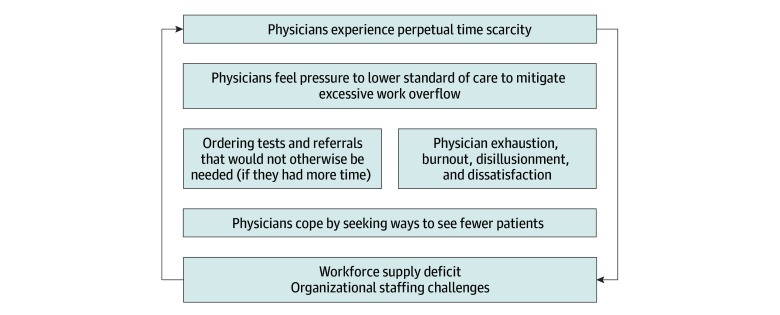
Time Allocation–Workload Expectation Mismatch

### Guilt, Exhaustion, and Burnout From Time Allocation and Work Expectations Mismatch

Participants felt that the way their employers allocated their work time was not well matched with others’ expectations of them.

How did we get to this point where we are expected to address all of these different things in such a short amount of time reasonably well and to the point where people are safe and taken care of? And…where we work so many hours that are unpaid because we’re documenting and doing all these other things throughout our entire lunch? We’re just expected to…give freely.

Another participant reported:

There’s no time allotted to do the follow-ups. “Abnormal results must be communicated in 48 hours,” and it’s like, “Okay, but the next 48 hours are fully booked-up with other things you’ve asked me to do. So, when do you expect me to do these 48-hour notifications? If you want me to do them, why don’t you set time aside for me to do them?”…It’s…feeling like it’s…very easy to ask me to do more things and not give me more time to do them in.

This mismatch between time allocation and work expectations created the experience of persistent time scarcity, which led to feelings of guilt, being constantly behind, exhaustion, and burnout.

It always feels like I’m letting someone down, either that person on the messages waiting a long time….or I’m with a patient and not paying attention to them. You feel bad because it’s not just you. You’re keeping the nurses behind. You’re keeping the office staff....It definitely makes you irritable….I come home…I don’t eat well, or I don’t exercise….You come home so exhausted.

### Sacrifices in Patient Care and Self-Care to Cope With and Mitigate Work Overflow

Most participants noted the inability to provide the quality of care they believe patients deserve without having their work overflow far outside normal business hours due to lack of time during their visits.

The kind of care that’s provided if you’re going to work only 50 or 60 hours a week is terrible care. I’m basically just funneling people to the hospital. The doctors that are compensated better are the ones that can see 50 patients in a day. It’s impossible to do a good job. So, either play the game and do a bad job, or you work all night.

Similarly:

I recently took Mondays off, so I don’t see patients on Mondays, but I always have between 3 and 7 hours of stuff to do to catch up.

In an attempt to balance quality of care and work overflow, participants described having to sacrifice aspects of patient care (ie, clear communication, patient education, relationship and trust building, and truly understanding patients’ problems and their social determinants of health) and self-care (ie, time to eat lunch, time to learn, and using their training and skills, such as in-office procedures or motivational interviewing). These physicians’ self-worth, well-being, and work satisfaction were intertwined with these sacrifices in patient care. They described spending quality time building relationships with, counseling, and educating patients as helping them sustain joy in their work and protect against burnout.

There’s something about feeling like you’re really able to spend time with the patient and get to know them so that you can actually know all the other medical facts that you need to know to care for them. Being able to do that is a way to inoculate ourselves against…burnout.

Participants noted that if they spent extra time during one encounter (eg, educating a patient about low-value testing), they would run behind the rest of the day, keeping other patients and their staff waiting and not having time to care for themselves or their families:

If we take the time and don’t [end the visit at the scheduled time], then there’s always a penalty that I end up having to pay.

### Unnecessary Testing and Referrals to Mitigate Work Overflow

To mitigate work overflowing in this manner, almost all the physicians admitted to ordering tests or referrals that they did not think they would have needed or wanted to order if they had more time to do more thorough evaluations. Some also felt that time constraints led them to send more patients to urgent care and emergency departments. These experiences support an overarching theme of patient care unnecessarily overflowing from the primary care setting into specialist, urgent care, and emergency settings due to time constraints. We have included representative quotations in Table 2.

**Table 2.** Low-Value Care Due to Constrained Time

Theme	Quotations
Unneeded testing	“I think that if I had more time to…get more history then [I] could tease out what might be the underlying thing or how much of it is a chronic thing that isn’t as urgent. That would help [me] figure out how much new information I needed, but sometimes it is this easier path to just try to get more information and analyze it later.”
	“There’s that, ‘Will you order things just to close it out, depending on the time?’ like where you’re pressed for time. I try not to, but I know it happens…if it’s a vague neurologic complaint and you’re like, ‘Oh, when was the last TSH? When was the last B_12_?’ Sometimes you’re just going to reorder them because…you don’t have time to look back.…If you don’t have the critical thinking time, it goes to that sort of shock and awe approach.”
	“When I don’t have time to critically think I shotgun more [order more tests] than [I] would just because…I don’t have time to re-reference this or take the time to gather all of the history that would talk myself out of feeling I need the lab.”
Unneeded referrals	“Diabetes, since they’ve clamped down on the good drugs, it’s become a nightmare. I love diabetes; I can manage it, no problem. I can’t do those prior authorizations…so I would definitely send my referral to [an] endocrinologist.”
	“The other place I find myself referring a lot for is a neuro eval[uation]…if I had time, I might be able to do…a thorough neuro exam.”
	“A really easy one is dermatology. Like when someone comes in and they have a skin lesion that I could biopsy, but we’ve already dealt with 5 other medical problems.”
	“Truthfully, if I had time, we should be doing EKGs in my clinic.…We don’t have time to do an EKG because we have to turn that room over….So it’s much easier to say, ‘Oh, you got to see cardiology.’”
Overflow of care to acute care settings, such as urgent care and emergency department	“When…you have a panel of 5000 patients and you have to get through all of them, and then do all of the charting, if someone needs to come in for a strep throat, do you think that my clinic does that? No, I send those people to urgent care because I don’t have time to deal with that. My patients on my panel: I don’t have time to see them when they’re sick. That’s crazy….Then [in] urgent care you go in and you have something that seems like it might be a little bit too much time, ‘Oh, you need to go to ER.’ I mean it literally just escalates.”

Abbreviations: EKG [ECG], electrocardiogram; ER, emergency room [department]; TSH, thyroid-stimulating hormone [thyrotropin].

### Feelings of Inadequacy and Dissatisfaction From Sacrifice of Personal Standards of Care 

The act of sacrificing personal standards of care (eg, ordering unneeded testing and referrals and not performing their own procedures) to minimize running behind and excessive overflow of work outside of normal business hours led many physicians to feel inadequate and unsatisfied.

I kind of leave with a feeling of inadequacy, feeling like…I’m not satisfying my oath to be the physician I want to be. Because…people [who] are going into primary care pride themselves in listening to the patient, getting the story….When you can’t do that, then…[it] feels like you’re not doing a good job.

### Workforce Supply Consequences

How do PCPs attempt to make their work more sustainable and fulfilling? Almost all the physicians shared strategies that involved reducing the number of patients they care for. This included participants who had already spent considerable effort optimizing their approaches to team-based care, administrative tasks, and/or electronic medical record work. Some looked for opportunities to decrease clinical time by taking administrative, leadership, or teaching roles; some transitioned to part-time designation; and others moved to other models of primary care with lower patient loads, such as concierge medicine or direct primary care. We have included representative quotations in Table 3.

**Table 3.** Current Strategies to Sustain a Primary Care Career

Theme	Quotations
Increasing nonclinical time (including taking leadership and teaching roles)	“My colleagues do it as well…we look for avenues where part of your clinical time will be bought by something else, not necessarily grants…but administrative roles, leadership roles, teaching roles, so that your clinical time and the number of patients that you care [for]…will decrease because the burnout is real, spending time charting in the evening, charting in bed on the weekends...”
	“Yes, [there are] definitely times where I’ve thought…‘This job is going to kill me.’ Not literally but you know, like this job is super stressful. Looking for things that will help take you out of clinic so that you have more of a balance which luckily I should start in about 3 weeks. So, I’m very much looking forward to that. I’m going from 70 to 50 [percent patient care time].”
Transitioning to other models of primary care	“I really wanted to maintain the…quality of what I did...But it became unmanageable. That’s why when I had this option to change practice, it was really at a point where I needed to do that. It was not sustainable. Something would have to give, and I’d have to compromise the way I managed patients.…I think the reason we continue to do this is because of the relationships we develop with people…if you take away the ability to develop relationships with your patients and you take away the autonomy of the physician, at some point, the physician’s going to decide, ‘Why am I going to continue doing this?’ So I think this type of practice that I have, having the ability to see fewer patients…for the physicians, that’s important. Those are the 2 key things: having time and having autonomy.”
Reducing to part-time clinical work	“Working long hours 5 days a week, that’s not sustainable, which is why none of the providers in my practice work full time. Most of us have at least a half day or a day off. Because I did try going full time the first few years and I just burned out.”
	“There are times when I think I need to go down to 4 days a week. My wife wants me to. Colleagues have done the same thing.”
	“I really want to make this sustainable and really want to be in this type of work for the long haul, especially FQHCs…but at this pace, I don’t think I realistically can for my own personal wellbeing…in the end, I probably would still stay in an FQHC but just cut down way, way part-time, like 2 days a week.…Everyone’s in the same boat because they either cut down…diversify their…work, or have left.”
	“I cut back my clinic hours....Everyone in our clinic has cut down.”

Abbreviation: FQHC, federally qualified health center.

In summary, the structural mismatch between time allocation and work expectations created a constant sense of time scarcity that led to negative emotions and forced these physicians to choose between sacrificing their standards of care and having their work overflow into their personal lives. They attempted to make their jobs more sustainable by seeking nonclinical roles, transitioning to other models of primary care, and reducing official work hours.

## Discussion

Study participants identified a mismatch between work expectations and the time given to them to complete that work. This finding is consistent with prior studies showing that PCPs routinely work between scheduled office visits^18^ and after hours (most notably completing tasks such as documentation in the electronic health record).^10,19^ Prior studies have also shown that the tasks currently expected from PCPs require more time than physicians have available.^20,21^ We clearly need to address the structural mismatch between our time allotment systems and work expectations in primary care.

The job demands–resources model of burnout helps us contextualize our findings and identify paths forward.^22^ The model differentiates between job demands that create physical and/or psychological costs for employees (in this study, PCPs) and the job resources that can buffer their impact, particularly when aligned with the demands faced.^23^ From this perspective, we can support employees by reducing job demands or increasing available job resources. In contrast, failing to intervene where there are excessive job demands can result in a health impairment process.^23^ Strain ultimately leads to negative outcomes, including psychological distress and burnout^23,24^—something evident in our findings regarding time constraints in primary care.^6,7,8,25^ Per the job demands–resources model, our participants detailed the physical and psychological costs and the negative impacts of strain resulting from excessive work demands.^22^ Due to the work expectation–time allocation mismatch, physicians felt like they had to choose between caring for patients to the standard they felt patients deserved and having their work overflow into their personal lives. No matter which option they “chose” (in reality, many reported sacrificing on both ends), they experienced negative emotions, most prominently guilt and exhaustion. Notably, their distress was caused by systemic and structural factors despite prominent individual effort to mitigate demands.^26,27^

This research helps contextualize prior evidence that time pressure leads to clinician stress, intent to leave, and burnout^8^ and that more time spent working after hours in the electronic medical record is associated with higher odds of exhaustion among PCPs.^9^ It also helps deepen our understanding of prior findings that demonstrated associations between shorter visit lengths and lower quality and/or value of care.^12,13,28^ Although prior studies have shown that physician gender is tied to patient expectations, documentation habits, and burnout,^29,30,31^ we did not find compelling differences in perspectives based on gender or caregiver status. The physicians in our study felt that they did not have time to care for patients in a way they could be proud of without sacrificing their personal time and well-being. We have created a system in which PCPs have no choice but to choose between their patients’ care and their own ability to sustainably work in the field.

Framed more broadly, these findings highlight that in the absence of organizations intervening to reduce job demands or sufficiently increase job resources, individual PCPs are resorting to job crafting. This is a proactive behavior by employees to change the level of job demands faced or job resources available to them to reflect their work-related needs and preferences.^32,33^

Job crafting by PCPs is systematically occurring in response to excessive work demands. Efforts are predominantly focused on reducing job demands by task crafting—changing the amount, scope, and type of clinical work undertaken (eg, taking leadership or teaching roles and transitioning to alternative care models). Importantly, the accompanying evidence of work withdrawal (ie, reducing hours) suggests that time constraints and their effects are beyond what individuals can personally respond to and absorb. In the absence of organizational support, PCPs are mitigating felt job demands by reducing clinical time. This finding is consistent with an observed trend of physicians nationwide seeking to work fewer scheduled hours to combat work overflow and burnout and obtain better work-life balance.^34,35^ The danger of further work intensification and extensification for those remaining is clear.

### Recommendations

Redesigning or transforming primary care without attending to scheduling risks perpetuating a foundational weakness in our current health care delivery systems.^36,37,38^ We suggest that policy leaders urgently look for and address any mismatch between their scheduling policies and the work expectations they demand of their PCPs. More specifically, we suggest leaders (1) increase slack in outpatient clinical scheduling by introducing more flexible time and (2) recognize their agency and responsibility to set care expectations that are realistic and match the time structures they create for their physicians. For example, employers may be able to expect physicians who have small panel sizes to care for patients comprehensively, but they should reevaluate their patient care expectations for physicians with thousands of patients, who realistically do not have enough time to care for patients in this manner.^21^ Health system leaders must also be conscious of their organizations’ tendencies to market idealized primary care to the community while not building strong support structures for their workforce to actually deliver this type of care. We urge leaders to set transparent and realistic expectations for their workforce and clearly communicate these expectations to patients, physicians, and managers.

### Limitations

This study has some limitations. We designed our study not to be generalizable to all PCPs in the US but rather as a deep exploration of human experience among a diverse group of physicians to assist in human-centered policymaking. Our recruitment process relied on physicians’ interest in the study, which may have overselected for physicians with strong opinions about the subject of time pressure. Our interviews were conducted during the COVID-19 pandemic, which likely affected our participants’ experiences and perspectives. Nonetheless, the challenges of time pressure in primary care existed long before this pandemic and remain persistent. Consequently, we believe our findings remain relevant to policymakers.

## Conclusions

In this qualitative study of time constraints in primary care, we found that the mismatch between current methods of time organization (ie, scheduling) and expectation setting is creating a scarcity mindset among our PCP workforce, leaving individual workers to cope with organizational and systemic issues, often without adequate resources. This issue is exacerbating our persistent quality of care and workforce supply problems. Organizations have an opportunity to support PCPs and enhance retention by increasing flexible time and rethinking workload expectations.

## Notes

Supplement 1. **eAppendix 1.** Mini-Z Burnout Survey **eAppendix 2.** Interview Guide **eAppendix 3.** Mini-Z Survey Results

Supplement 2.
Data Sharing Statement
