# Supplementary material for: Primary Care Physicians’ Experiences With and Adaptations to Time Constraints
Source: JAMA Netw Open. 2024 Apr 30;7(4):e248827. doi: 10.1001/jamanetworkopen.2024.8827 (PMC11061766; doi:10.1001/jamanetworkopen.2024.8827)
Supplement: Supplement 1. — eAppendix 1. Mini-Z Burnout Survey eAppendix 2. Interview Guide eAppendix 3. Mini-Z Survey Results [file jamanetwopen-e248827-s001.pdf]

Supplementary Online Content

Nguyen M-LT, Honcharov V, Ballard D, Satterwhite S, McDermott AM, Sarkar U. Primary care physicians' experiences with and adaptations to time constraints. *JAMA Netw Open*. 2024;7(4):e248827. doi:10.1001/jamanetworkopen.2024.8827

eAppendix 1. Mini-Z Burnout Survey
eAppendix 2. Interview Guide
eAppendix 3. Mini-Z Survey Results

This supplementary material has been provided by the authors to give readers additional information about their work.

eAppendix 1. Mini-Z Burnout Survey

Answer the following questions as truthfully as possible to determine your workplace stress levels and how they measure up against others in your field. There are two sections of questions in this survey about your experience with burnout and your practice environment.

**Mini Z burnout survey**	
**Name:**	**Role:**
**Team/department:**	**Date of survey:**
For questions 1-10, please choose the answer that best describes your experience with burnout. Please circle your answers.	

1. Overall, I am satisfied with my current job:	1 Strongly disagree	2 Disagree	3 Neutral	4 Agree	5 Strongly Agree
2. I feel a great deal of stress because of my job:	1 Strongly disagree	2 Disagree	3 Neutral	4 Agree	5 Strongly Agree

3. Using your own definition of "burnout," please circle one of the answers below:

   a. I enjoy my work. I have no symptoms of burnout.
   b. I am under stress, and don't always have as much energy as I did, but I don't feel burned out.
   c. I am definitely burning out and have one or more symptoms of burnout, e.g., emotional exhaustion.
   d. The symptoms of burnout that I am experiencing won't go away. I think about work frustrations a lot.
   e. I feel completely burned out. I am at the point where I may need to seek help.

4. My control over my workload is:	1 Poor	2 Marginal	3 Satisfactory	4 Good	5 Optimal
5. Sufficiency of time for documentation is:	1 Poor	2 Marginal	3 Satisfactory	4 Good	5 Optimal
6. Which number best describes the atmosphere in your primary work area?	1 Calm	2	3 Busy, but reasonable	4	5 Hectic, chaotic
7. My professional values are well aligned with those of my department leaders:	1 Strongly disagree	2 Disagree	3 Neither agree nor disagree	4 Agree	5 Strongly Agree

8. The degree to which my care team works efficiently together is:	1<br>Poor	2<br>Marginal	3<br>Satisfactory	4<br>Good	5<br>Optimal

9. The amount of time I spend on the electronic health record (EHR) at home is:	1<br>Excessive	2<br>Moderately high	3<br>Satisfactory	4<br>Modest	5<br>Minimal/none
10. My proficiency with EHR use is:	1<br>Poor	2<br>Marginal	3<br>Satisfactory	4<br>Good	5<br>Optimal

11. Tell us more about your stresses and what we can do to minimize them (optional):

Your clinical practice

Answer the following questions as truthfully as possible to determine your workplace stress levels and how they measure up against others in your field.

For the following, please tell us about yourself and your practice. Please fill in the blanks.	
Are you: __ MD/DO __ NP __ PA __ Other (specify):____________________	
Specialty:	Practice name:
City of practice:	State of practice:
Practice size (# physician FTEs):	Are you: __ Employed __ Owner
Practice type: __ VA __ Non-VA	Practice specialty:
EHR vendor (optional):	
For the following, please choose the answer that best describes you.	
Where do you spend the majority of your clinical time? __ Inpatient __ Outpatient	
Please tell us the number of years in your current role: ____________	
Gender (optional): __ Female __ Male	
Race (optional): __ Black or African American __ Asian __ Native American<br>__ Native Hawaiian or Other Pacific Islander __ White	
Ethnicity (optional): __ Latino/Hispanic __ Not Latino/Hispanic __ Prefer not to answer	

© 2024 Nguyen M-LT et al. *JAMA Network Open.*

eAppendix 2. Interview Guide

Thank you for taking time to fill out the surveys. I'd love to learn more about your experience with time and how you manage time as it relates to caring for your patients on a typical clinic day. This is a completely judgment-free space. There are no wrong answers here; we are really just interested in anything you have to share. This interview will take about 30 minutes.

- In general, how do you manage time during a typical clinic day?
  - **Probe:** How does time affect how you practice medicine and care for your patients?
  - **Probe:** How do you deal with time constraints?

- Are there certain portions of a clinical visit that you try to never leave out, even if you are short on time? Could you tell me more about this?
  - **Probe:** Are there certain portions of a clinical visit that you leave out more often than others when time is short? How do you make those decisions?
  - **Probe:** How does this decision-making process make you feel?

- Do you feel you have enough time to critically think about your patients' care?
  - **Probe:**
    - *If yes:* when do you usually do this? (i.e. during the clinical visit in front of the patient, after the clinical visit, while writing the note)
    - *If no:* how do you cope with that?

- Do you ever order tests that you feel would be unneeded if you had more time?
  - **Probe:** How about referrals? Medications?

- Could you tell me about a scenario or scenarios where a patient's care clearly did not fit within the appointment time?
- Could you tell me about a time where you running low on time, but chose not to cut a visit short or interrupt a patient, led to a change in your management for that patient?

- Can you tell me about a scenario where time management issues lead you to feel negatively about yourself, your patient, or your profession?
  - **Probe:** Does this happen often?
  - **Probe:** How does this affect your desire to work in primary care?
  - **Probe:** How does this affect your mental health?

- CMS changed their billing structure to include an option to bill by time starting in January 2021. Did you know about this? Has this affected the way you care for your patients / Do you think it will?
  - **Probe:**
    - *If yes*, how so?
    - *If no*, how come?

- If you were all-powerful for a day, if resources were not an issue, how would you imagine an ideal clinic day?
  - **Probe:** How would you change how time is allocated and used during a clinic day?

© 2024 Nguyen M-LT et al. *JAMA Network Open.*

eAppendix 3. Mini-Z Survey Results

Mini-Z Survey Results			
		**Median Score (Range)**	**Survey Answer Associated with Median Score**
	Overall Score	30 (21-37)	-
**Question**			
1	Overall, I am satisfied with my current job:	4 (1-4)	Agree
2	I feel a great deal of stress because of my job:	4 (1-5)	Agree
3	Using your own definition of burnout please select one of the answers below:<br><br>1. I enjoy my work. I have no symptoms of burnout.<br>2. I am under stress, and don’t always have as much energy as I did, but I don’t feel burned out.<br>3. I am definitely burning out and have one or more symptoms of burnout, e.g., emotional exhaustion.<br>4. The symptoms of burnout that I am experiencing won’t go away. I think about work frustrations a lot.<br>5. I feel completely burned out. I am at a point where I may need to seek help.	3 (2-4)	-
4	My control over my workload is:	2 (1-5)	Marginal
5	Sufficiency of time for documentation is:	2 (1-5)	Marginal
6	Which number best describes the atmosphere in your primary work area?	4 (1-5)	In-between “Busy, but reasonable” and “Hectic, chaotic”
7	My professional values are well aligned with those of my department leaders:	3 (1-5)	Neither agree nor disagree
8	The degree to which my care team works efficiently together is:	3 (1-5)	Satisfactory
9	The amount of time I spend on the electronic health record (EHR) at home is:	2 (1-5)	Moderately high
10	My proficiency with EHR use is:	4 (2-5)	Good

© 2024 Nguyen M-LT et al. *JAMA Network Open.*
