# Supplementary material for: Primary Care Physicians’ Experiences With and Adaptations to Time Constraints
Source: JAMA Netw Open. 2024 Apr 30;7(4):e248827. doi: 10.1001/jamanetworkopen.2024.8827 (PMC11061766; doi:10.1001/jamanetworkopen.2024.8827)
Supplement: Supplement 2. — Data Sharing Statement [file jamanetwopen-e248827-s002.pdf]

Data Sharing Statement

Nguyen. Primary Care Physicians’ Experiences With and Adaptations to Time Constraints. *JAMA Netw Open*. Published April 30, 2024. doi:10.1001/jamanetworkopen.2024.8827

Data
Data available: Yes
Data types: Deidentified participant data
How to access data: De-identified data will be made available upon reasonable request to the corresponding author.
When available: beginning date: 11-13-2023

Supporting Documents
Document types: None

Additional Information
Who can access the data: De-identified data will be made available upon reasonable request to the corresponding author.
Types of analyses: The de-identified data will be made available for analyses that are directly related to the objectives of the current study. We will strive to accommodate requests that contribute meaningfully to the understanding and advancement of the study's subject matter.
Mechanisms of data availability: De-identified data will be made available without investigator support. A data access agreement may be provided before access to data is granted.
Any additional restrictions: N/A
